# An Individualized Recommendation for Controlled Ovary Stimulation Protocol in Women Who Received the GnRH Agonist Long-Acting Protocol or the GnRH Antagonist Protocol: A Retrospective Cohort Study

**DOI:** 10.3389/fendo.2022.899000

**Published:** 2022-07-22

**Authors:** Ming-Xing Chen, Xiang-Qian Meng, Zhao-Hui Zhong, Xiao-Jun Tang, Tian Li, Qian Feng, Enoch Appiah Adu-Gyamfi, Yan Jia, Xing-Yu Lv, Li-Hong Geng, Lin Zhu, Wei He, Qi Wan, Yu-Bin Ding

**Affiliations:** ^1^ Department of Obstetrics and Gynecology, Women and Children’s Hospital of Chongqing Medical University, Chongqing, China; ^2^ Joint International Research Laboratory of Reproduction and Development of the Ministry of Education of China, School of Public Health, Chongqing, China; ^3^ Reproductive Medical Center, Chengdu Xinan Gynecological Hospital, Chengdu, China; ^4^ Department of Epidemiology, School of Public Health and Management, Research Center for Medicine and Social Development, Innovation Center for Social Risk Governance in Health, Chongqing Medical University, Chongqing, China; ^5^ The Department of Reproductive Medicine, The First Affiliated Hospital of Chongqing Medical University, Chongqing, China; ^6^ Department of Gynecology, Chongqing Hospital of Traditional Chinese Medicine, Chongqing, China; ^7^ Infertility and Infertility Center, Chengdu Jinjiang Hospital for Women‘s and Children’s Health, Chengdu, China; ^8^ Reproductive Medical Center, Southwest Hospital, Army Medical University, Third Military Medical University, Chongqing, China

**Keywords:** GnRH agonist long-acting protocol, GnRH antagonist protocol, live birth rate, ovarian reserve, body mass index

## Abstract

**Background:**

The GnRH agonist long-acting protocol and GnRH antagonist protocol are widely used in ovarian stimulation. Which protocol eliciting higher live birth rate for IVF/ICSI patients with different ages, different ovarian reserves and different body mass index (BMI) has not been studied. However, among these protocols, the one that elicits higher live birth in IVF/ICSI patients with different ages, ovarian reserves and body mass indexes (BMI) has not been identified.

**Methods:**

This was a retrospective cohort study about 8579 women who underwent the first IVF-ET from January, 2018 to August, 2021. Propensity Score Matching (PSM) was used to improve the comparability between two protocols.

**Results:**

After PSM, significant higher live birth rates were found in the GnRH agonist long-acting protocol compared to GnRH antagonist protocol (44.04% vs. 38.32%) (p<0.001). Stratified analysis showed that for those with AMH levels between 3 ng/ml and 6 ng/ml, with BMI ≥ 24 kg/m^2^ and were aged ≥ 30 years old, and for those women with BMI < 24kg/m^2^ and were aged ≥30 years whose AMH levels were ≤ 3ng/ml, the GnRH agonist long-acting protocol was more likely to elicit live births [OR (95%CI), 2.13(1.19,3.80)], [OR (95%CI), 1.41(1.05,1.91)]. However, among women with BMI ≥ 24kg/m^2^ and were aged ≥30 years whose AMH levels were ≤ 3ng/ml, the GnRH agonist long-acting protocol had a lower possibility of eliciting live births [OR (95%CI), 0.54(0.32,0.90)]. Also, among women with AMH levels between 3 ng/ml and 6 ng/ml, with BMI ≥ 24 kg/m^2^ and with age < 30 years and for those with AMH levels between 3 ng/ml and 6 ng/ml, regardless of age, and with BMI<24kg/m2,, the possibility of live births was similar between the two protocols [OR (95%CI), 1.06(0.60,1.89)], [OR (95%CI), 1.38(0.97,1.97)], [OR (95%CI), 0.99(0.72,1.37)]. Among the women with AMH levels ≤ 3 ng/ml and with were aged < 30years, regardless of BMI, the possibility of live birth was similar between the two protocols [OR (95%CI), 1.02(0.68,1.54)], [OR (95%CI), 1.43(0.68,2.98)]. Moreover, among women with AMH levels ≥ 6ng/ml, the possibility of live birth was similar between the two protocols [OR (95%CI),1.42(0.75,2.69)], [OR (95%CI),1.02(0.19,5.35)], [OR (95%CI), 1.68(0.81,3.51)], [OR (95%CI), 0.51(0.10,2.55)].

**Conclusions:**

The suitability of the GnRH agonist long-acting protocol or GnRH antagonist protocol to infertility patients is dependent on specific biological characteristics of the patients.

## Introduction

*In vitro* fertilization and embryo transfer (IVF-ET) is the most commonly patronized treatment option for women experiencing infertility. This is attributable to the increase in pregnancy rates of patients undergoing IVF-ET. A key to the improvement in pregnancy rate is the application of the controlled ovarian stimulation (COS) protocols (1, 2). Among the COS protocols that have been developed are the gonadotropin-releasing hormone (GnRH) agonist long protocol and the GnRH antagonist protocol (2, 3). The GnRH agonist long-acting protocol is one of the mainstream protocols of COS in China because of its advantages such as effectively improving endometrial receptivity and increasing the clinical pregnancy rate of fresh IVF cycles (4, 5). The GnRH antagonist protocol, on the other hand, is widely used because of its shorter duration of stimulation and its association with a low incidence of ovarian hyperstimulation syndrome (OHSS) (5–7).

Since both protocols are advantageous to some extent, clinicians have become indecisive about which one to fully rely upon. Previous studies that compared both protocols on live birth rates yielded seemingly conflicting findings. Yang et al. reported (8) that live birth rate, clinical pregnancy rate and implantation rate of the GnRH agonist long-acting protocol are significantly higher than those of the antagonist protocol. However, Wang et al. found (9) that there is no significant difference in live birth rate between both protocols in patients with normal ovarian reserves. Li et al. (10) observed that in patients with poor ovarian response, the GnRH agonist long-acting protocol is associated with higher live birth rates than the GnRH-antagonist protocol. These seemingly conflicting reports, together with the confounding factors such as variation in the basic characteristics of women, make it difficult to decide on which of the two protocols is optimal for IVF women. Hence, it is necessary to implement individualized COS protocols in accordance with the specific characteristics of the patients.

An important clinical feature of female infertility is ovarian reserve, which is also a crucial factor used in selecting the most appropriate COS protocol (11–13). Several studies have shown that AMH is a reliable marker of ovarian reserve (14–18), and has a significant correlation with age (19, 20). Due to this, AMH, combined with age, is commonly used to evaluate ovarian reserve in clinical practice.

It has been found that increased body mass index (BMI) affects the success of IVF (21, 22) as well as live births following IVF (23). Also, it has been observed that serum AMH is positively correlated with BMI in normal weight women with normal ovarian reserve (24). However, in women with polycystic ovary syndrome (PCOS), serum AMH was observed to correlate negatively with BMI (25). These findings indicate that BMI and AMH serum levels should be taken into account when establishing an individualized COS protocol. Thus, in this study, we retrieved the data of infertile women who had been exposed to the GnRH agonist long-acting protocol or the antagonist protocol, and assessed their live birth rate by combining the basic characteristics: age, BMI and AMH levels. Our findings would provide reference for clinical guidance and treatment of female infertility.

## Material and Methods

### Participants

**Figure 1:**
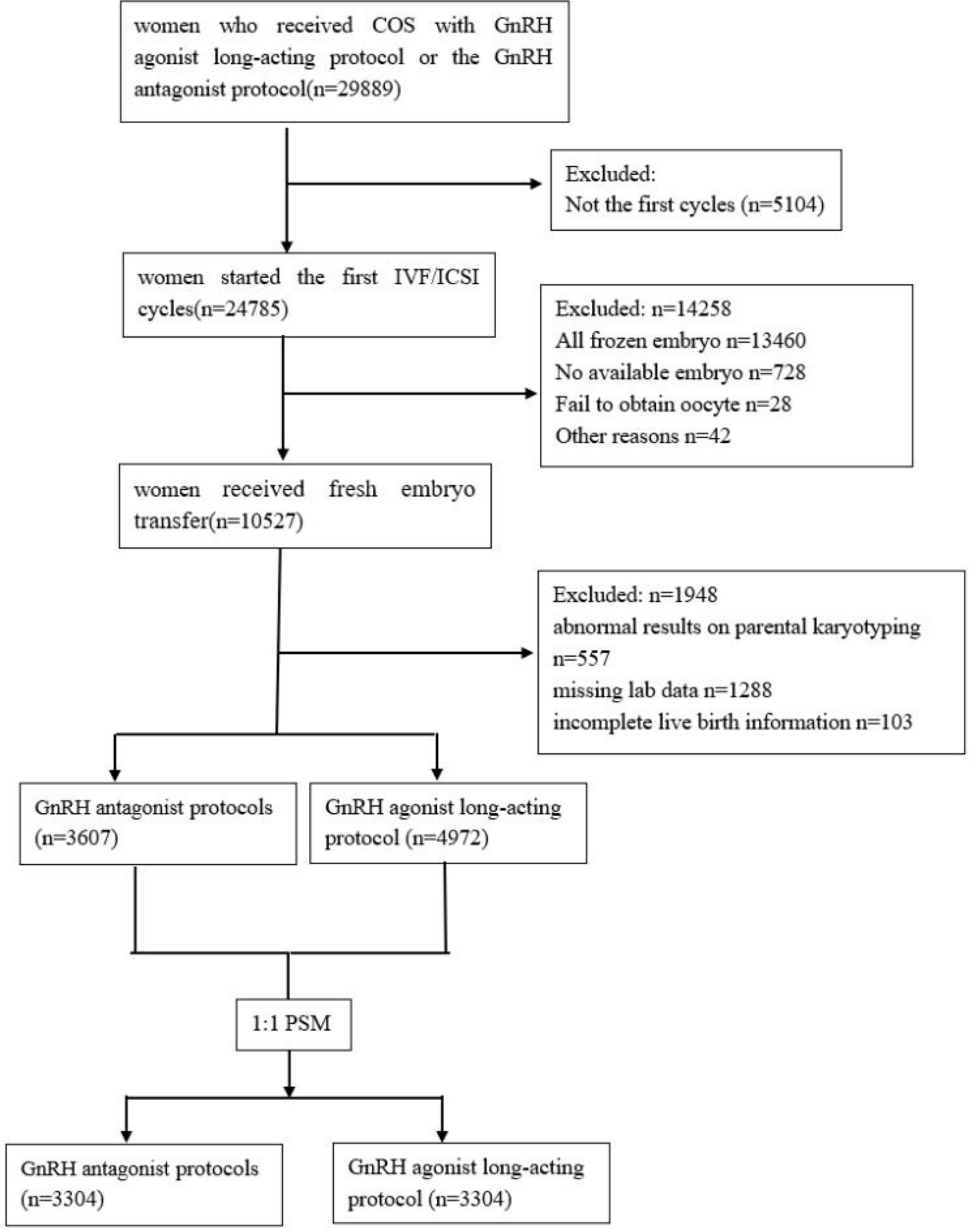
Flow chart.

Women who had undergone their first IVF/ICSI cycles between January, 2018 and August, 2021 at the Chengdu Xinan Gynecology Hospital and Chengdu Jinjiang Hospital for Women’s and Children’s Health were retrospectively identified in the institutional database. Only women who received COS with GnRH agonist long-acting protocol or the GnRH antagonist protocol and received fresh embryo transfer were included in this study. Exclusion criteria were abnormal results on parental karyotyping, missing lab data, and incomplete live birth information. Patients' flow chart detailing the whole process is shown in **Figure 1**.

### GnRH Agonist Long-Acting Protocol

Each woman received a GnRH agonist (Diphereline, 3.75mg, Beaufort-Ipson, France) on the 2nd to 4th day of menstruation (follicular phase). Serum levels of sex hormones and ultrasound assessment of developing follicles were monitored on the 28th to the 35th day after GnRH agonist administration. The following criteria were used: (a) endometrial thickness < 5mm, (b) estradiol (E2) < 50pmol/L, luteinizing hormone (LH) < 5IU/L follicle-stimulating hormone (FSH) <5 IU/L, progesterone (P) of <1 ng/ml, (c) no functional cyst, (d) follicle size 3–5 mm under ultrasound. In accordance with the patient’s age, BMI, antral follicle number (AFC) and AMH levels, we determined the initial dose of gonadotropin (Gn) that could control ovulation. The dosage was adjusted continually according to the patient’s ovarian reaction and follicular growth. 250 µg of recombinant human chorionic gonadotropin (rhCG, Merck Schlano, Germany) were given to each woman until two to three ovarian follicles were, at least, 17–18 mm in diameter. Oocyte retrieval was performed 36 hours post-hCG injection.

### GnRH Antagonist Protocol

In accordance with the patient’s age, BMI, antral follicle number (AFC) and AMH levels, recombinant FSH 100 ~ 300 IU/d (rFSH, Gonal-F, Merck Serono S.A., Switzerland) administration was done from the 2nd to the 4th day of the menstrual cycle. This was followed by Gn administration. The Gn dosage was adjusted as the follicles developed. A daily dose of 0.25 mg GnRH-ant (Ganerik acetate, Merck Serono, Switzerland) was started either on the 6th day of rFSH stimulation until the hCG injection or when the dominant follicle’s diameter was ≥ 12-14 mm. The induction of ovulation was performed by administering the women with 250 µg of rhCG (Merck Schlano, Germany) or with the 0.2 mg of Decapeptyl either alone or in combination with, 2000 IU of urinary hCG [Merck Schlano]). This was done during the period when two to three ovarian follicles were, at least, 17–18 mm in diameter. Oocyte retrieval was performed 36 hours after the ovulation induction.

### Embryo Transfer and Luteal Support

On the 3rd to 5th day after fertilization, 1 to 2 of grade I-II high-quality embryos were selectively transferred. Embryo grading was done in accordance with the proceedings of the Istanbul consensus (26). The luteal phase support was started on the day when the oocytes were retrieved with 200 mg intravaginal progesterone soft capsules for 8 hours/times. 20mg of dydrogesterone (Dupbaston, Dutch) was taken twice on each day.

### Outcome Measures

The primary outcome measure was the live birth which was defined as the delivery of any living baby at or after 28 weeks of pregnancy during the first embryo transfer. Live birth rate = number of live birth cycles/number of embryo transfer cycles. The secondary outcomes were biochemical pregnancy rate, clinical pregnancy rate, incidence of ovarian hyperstimulation syndrome (OHSS), number of retrieved oocytes, number of metaphase II (MII) oocytes, and number of 2 pronuclear (2PN) embryos. The biochemical pregnancy was defined as the serum β-HCG>25U/L 14 days after embryo transfer Clinical pregnancy, defined as the presence of gestational sac or fetal heart, was confirmed with transvaginal ultrasound 28 days after embryo transfer. The OHSS was defined according to the Golan et al’ criteria (27).

### Statistical Analysis

Propensity Score Matching (PSM) was used in data analysis to balance the baseline and improve the comparability between GnRH agonist long-acting protocol group and GnRH antagonist protocol group. The variables in PSM model included female age, BMI, duration of infertility, type of infertility, basal sex hormone (E2, P, FSH, LH), AFC, AMH, insemination methods, the number of good quality embryos transferred and the type of embryos transferred. A 1:1 nearest neighbor matching method with caliper (0.1) was used to match data between groups.

Continuous variables are expressed as mean ± SD or median (IQR); and Categorical variables are expressed as number (n) and percentage (%). Normality was checked through Shapiro-Wilk normality test. Mann-Whitney U test or Student’s t-tests were used for continuous variables and the Chi-square test was used for categorical variables.

Multivariate logistic regression analysis was performed to compare the live birth rates between the two protocols. Additional analyses were performed after stratification of the participants by age (age<30 years vs age≥30 years) (28), BMI (BMI<24 kg/m^2^ vs BMI≥24 kg/m^2^), AMH levels (AMH ≤ 3ng/ml vs 3ng/ml<AMH<6ng/ml vs AMH≥6ng/ml) (29) and also after combining the above three parameters. All analyses were performed using the Statistical Package for the Social Sciences (Version 25.0; SPSS, Chicago, IL). P <0.05 was used to indicate a significant statistical difference.

## Results

### Demographic, Cycle Characteristics and Pregnancy Outcomes Calculated Without Specific Stratification

The demographic characteristics, cycle characteristics and pregnancy outcomes of the study participants before and after PSM are shown in **Tables 1**, **2**. Before PSM, a total of, 8579 cycles were included in this study. Significant differences in the comparison of baseline characteristics were observed between two groups in age, BMI, AMH, AFC, basal FSH, basal LH, basal E2, basal P, Gn dose, duration of Gn, number of good quality embryos transferred., 4972 of the cycles used the GnRH agonist long-acting protocol and generated 45.09% of live birth rate while, 3607 of the cycles used the GnRH antagonist protocol and generated 38.70% of live birth rate. After 1:1 matching, a total of, 6608 cycles were analyzed in this study. There were no significant differences in age, BMI, basal FSH, basal LH, and the number of good quality embryos transferred between the two groups. However, the GnRH agonist long protocol group still received a higher gonadotropin dosage (1875IU vs, 1800IU) and longer gonadotropin exposure duration (12 vs 9) than the antagonist protocol group., 3304 of the cycles used the GnRH agonist long-acting protocol and generated 44.04% of live birth rate while, 3304 of the cycles used the GnRH antagonist protocol and generated 38.32% of live birth rate. The live birth rate of the GnRH agonist long-acting protocol group was significantly higher than that of the GnRH antagonist protocol group before and after matching(P<0.001).

**Table 1:** Comparison of baseline parameters between the GnRH agonist long-acting protocol and GnRH antagonist protocol and after PS matching.

	Before matching			after matching		
	GnRH antagonist	GnRH agonist	P value	GnRH antagonist	GnRH agonist	P value
NO. of cycles	3607	4972		3304	3304	
Female age (year)	30.59 ± 4.18	30.39 ± 3.75	0.021*	30.58 ± 4.17	30.49 ± 3.78	0.884
BMI (kg/m2)	22.28 ± 3.20	21.92 ± 3.01	< 0.001*	22.15 ± 3.13	22.10 ± 3.10	0.523
^a^Duration of infertility (years)	3 (2,5)	3 (2,5)	0.107	3 (2,5)	3 (2,5)	0.809
^a^Basal FSH (MIU/mL)	7.54 (6.49,8.77)	7.45 (6.35,8.70)	0.002*	7.54 (6.49,8.77)	7.52 (6.46,8.84)	1
^a^Basal LH (MIU/mL)	3.96 (2.95,5.30)	3.87 (2.84,5.17)	0.001*	3.88 (2.89,5.12)	3.92 (2.90,5.27)	0.389
^a^Basal E2 (p g/mL)	44 (34,57)	47 (36,62)	< 0.001*	44 (34,57)	47 (35,61)	< 0.001*
^a^Basal P (ng/mL)	0.56 (0.38,0.80)	0.58 (0.39,0.84)	< 0.001*	0.56 (0.38,0.80)	0.58 (0.39,0.84)	< 0.001*
^a^AFC	15 (10,21)	14 (11,18)	< 0.001*	14 (10,20)	15 (11,19)	0.002*
^a^AMH (ng/mL)	3.28 (1.98,5.21)	3.11 (2.31,4.17)	< 0.001*	3.04 (1.90,4.82)	3.25 (2.38,4.38)	< 0.001*
^a^Total dose of Gn (IU)	1800 (1425,2100)	1875 (1500,2325)	< 0.001*	1800 (1488,2175)	1875 (1500,2325)	< 0.001*
^a^Duration of Gn (d)	9 (8,10)	12 (11,13)	< 0.001*	9 (8,10)	12 (10,13)	< 0.001*
Cause of infertility			0.872			0.755
Tubal factor	2036 (56.45%)	2868 (57.68%)		1863 (56.39%)	1894 (57.32%)	
Pelvic and uterine factor	309 (8.57%)	407 (8.19%)		290(8.78%)	266 (8.05%)	
PCOS	200 (5.54%)	256 (5.15%)		190 (5.75%)	176 (5.33%)	
male factor	593 (16.44%)	815 (16.39%)		531 (16.07%)	549 (16.62%)	
female and male factors	185 (5.13%)	245 (4.93%)		174 (5.27%)	161 (4.87%)	
Other causes	284 (7.87%)	381 (7.66%)		256 (7.75%)	258 (7.81%)	
Infertility type (n, %)			0.592			1
Primary infertility	1837 (50.93%)	2503 (50.34%)		1665 (50.39%)	1665 (50.39%)	
Secondary infertility	1770 (49.07%)	2469 (49.66%)		1639 (49.61%)	1639 (49.61%)	
Fertilization method (n, %)			0.572			0.899
IVF	2931 (81.26%)	4064 (81.74%)		2689 (81.39%)	2693 (81.51%)	
ICSI	676 (18.74%)	908 (18.26%)		615 (18.61%)	611 (18.49%)	
No. of embryos transferred (n, %)			0.866			0.734
1	725 (20.10%)	992 (19.95%)		658 (19.92%)	647 (19.58%)	
2	2882 (79.90%)	3980 (80.05%)		2646 (80.08%)	2657 (80.42%)	
Embryo type (n, %)			0.301			0.933
Day3	2648 (73.41%)	3600(72.41%)		2429 (73.52%)	2432 (73.61%)	
Day5	959 (26.59%)	1372 (27.59%)		875 (26.48%)	872 (26.39%)	
No. of good quality embryos transferred (n, %)			0.001*			0.936
0	1118 (31.00%)	1371 (27.57%)		987 (29.87%)	976 (29.54%)	
1	1153 (31.97%)	1732 (34.84%)		1087 (32.90%)	1085 (32.84%)	
2	1336 (37.04%)	1869 (37.59%)		1230 (37.23%)	1243 (37.62%)	

BMI, body mass index; AFC, antral follicular count; AMH, anti-Müllerian hormone; FSH, follicle stimulating hormone; LH, luteinizing hormone; E2, estradiol; P, Progesterone; Gn, Gonadotropin; ICSI, intracytoplasmic single sperm injection; IVF, in vitro fertilization;

Data are presented as mean ± SD, median (IQR) and n (%).

Chi-square test, Mann-Whitney U test and Student’s t-tests were used for the preliminary comparison between the two groups.

a Cited as median (IQR).

^*^Statistically significant (P < 0.05).

**Table 2:** Comparison of clinical outcomes between the GnRH agonist long-acting protocol and GnRH antagonist protocol and after PS matching.

	Before matching			after matching		
	GnRH antagonist	GnRH agonist	P value	GnRH antagonist	GnRH agonist	P value
Number of retrieved oocytes	9.30 ± 4.37	9.73 ± 4.22	<0.001*	9.16 ± 4.28	9.69 ± 4.22	<0.001*
Number of MII oocytes	8.17 ± 3.99	8.57 ± 3.90	<0.001*	8.05 ± 3.91	8.51 ± 3.90	<0.001*
Number of 2PN embryos	6.13 ± 3.37	6.14 ± 3.29	0.941	6.05 ± 3.32	6.07 ± 3.27	0.802
^b^ OHSS rate	2.13% (77/3607)	4.42% (220/4972)	<0.001*	1.91% (63/3304)	4.57% (151/3304)	<0.001*
^b^ Live birth	38.70% (1396/3607)	45.09% (2242/4972)	<0.001*	38.32% (1266/3304)	44.04% (1455/3304)	<0.001*
^b^ biochemical pregnancy	55.78% (2012/3607)	61.38% (3052/4972)	<0.001*	55.75% (1842/3304)	60.90% (2012/3304)	<0.001*
^b^ Clinical pregnancy	47.91% (1728/3607)	53.74% (2672/4972)	<0.001*	47.79% (1579/3304)	53.03% (1752/3304)	<0.001*
^b^ ectopic pregnancy	3.99% (69/1728)	1.46% (39/2672)	<0.001*	4.12% (65/1579)	1.43% (25/1752)	<0.001*
^b^ Early Miscarriage	13.54% (234/1728)	13.14% (351/2672)	0.699	14.06% (222/1579)	13.87 (243/1752)	0.875
^b^ Late Miscarriage	2.26% (39/1728)	1.72% (46/2672)	0.208	2.28% (36/1579)	1.94% (34/1752)	0.495

MII, metaphase II; 2PN, 2 pronuclear; OHSS, ovarian hyperstimulation syndrome;

Data are presented as mean ± SD and n (%).

Student’s t-tests and Chi-square test were used for comparison of clinical outcomes between the two groups.

^*^Statistically significant (P < 0.05).

After matching, the number of oocytes retrieved (9.69 ± 4.22 vs 9.16 ± 4.28), the mature eggs number (8.51 ± 3.90 vs 8.05 ± 3.91), the biochemical pregnancy rate (60.90% vs 55.75%), the clinical pregnancy rate (53.03% vs 47.79%) and the incidence of OHSS (4.57% vs 1.91%) were higher in the GnRH agonist long-acting protocol group than in the antagonist protocol group. Nonetheless, the ectopic pregnancy rates (1.43% vs 4.12%) in the GnRH agonist long-acting protocol group were significantly lower than those of the GnRH antagonist protocol group. There was no significant difference in the two-Pro-Nuclei (2PN) fertilized eggs number (6.07 ± 3.27 vs 6.05 ± 3.32), early miscarriage (13.87% vs 14.06%) and late miscarriage rate (1.94% vs 2.28%) between two groups (**Table 2**).

### Live Birth Measured With Stratification Analysis Using Multivariate Logistic Regression

Before and after matching, and after adjusting for potential confounding factors (such as age, BMI, AMH, AFC, basal FSH, basal LH, basal E2, basal P, Gn dose, duration of Gn, number of good quality embryos transferred), the multivariate logistic regression analysis showed that the GnRH agonist long-acting protocol was associated with a higher possibility of having live birth than that of the GnRH antagonist protocol [OR (95%CI), 1.25(1.01,1.53)], P<0.001; [OR (95%CI), 1.20(1.00,1.43)], P=0.002 (**Table 3**).

**Table 3:** Comparison of live birth rate of the GnRH agonist long-acting protocol and GnRH antagonist protocol using multivariable logistic regression analysis in subgroup women with different BMI, AMH and age and after PS matching. (the GnRH antagonist protocol as a reference).

	Before matching		after matching	
	Adjusted OR (95% CI)	P	Adjusted OR (95% CI)	P
Total	1.24 (1.10,1.40)	<0.001	1.24 (1.08,1.42)	0.002
Age (year)				
<30	1.35 (1.12,1.61)	0.001*	1.25 (1.01,1.53)	0.036*
≥30	1.14 (0.973,1.33)	0.105	1.20 (1.00,1.43)	0.047*
BMI (kg/m2)				
<24.0	1.30 (1.13,1.49)	<0.001*	1.28 (1.10,1.50)	0.002*
≥24.0	1.11 (0.88,1.41)	0.382	1.17 (0.90,1.52)	0.249
AMH (ng/ml)				
AMH ≤ 3	1.12 (0.94,1.34)	0.205	1.12 (0.92,1.38)	0.264
3 <AMH<6	1.31 (1.09,1.57)	0.004*	1.24 (1.02,1.52)	0.035*
AMH≥6	1.21 (0.83,1.76)	0.314	1.41 (0.92,2.15)	0.115

CI, confidence interval

adjusting for confounders of female age, female BMI, AMH, AFC, basal E2, basal FSH, basal LH, basal P, number of good quality embryos, total dose of Gn, duration of Gn.

^*^Statistically significant (P < 0.05).

To find the live birth rate of the GnRH agonist long or antagonist protocols in patients with different characteristics, we carried out a further analysis by stratifying the patients according to their ages, BMIs and AMH levels. After matching, the multivariate logistic regression analysis showed a significantly higher possibility of having live births of each layer stratified by age in the GnRH agonist long protocol group than in the GnRH antagonist protocol group [OR (95%CI), 1,24(1.10,1.40)], [OR (95%CI), 1.24(1.08,1.42)]. For women with BMI <24kg/m^2^, the GnRH agonist long-acting protocol was associated with a higher opportunity of getting live births [OR (95%CI), 1.28(1.10,1.50)]; for women with overweight (BMI≥24kg/m^2^), the two protocols had similar live birth rates [OR (95%CI),1.17(0.90,1.52)]. When the population was stratified by AMH, for women with normal ovarian reserves (3ng/ml<AMH<6ng/ml), we found a significantly higher possibility of live birth in the GnRH agonist long protocol group than in the GnRH antagonist protocol group [OR (95%CI), 1.24(1.02,1.52)]; Among women with AMH≥ 3ng/ml or AMH≥ 6ng/ml, the chances of getting live births were similar between the two groups. [OR (95%CI),1.12(0.92,1.38)], [OR (95%CI), 1.41(0.92,2.15)] (**Table 3**).

After matching, the study population was divided into 12 groups according to the combination of AMH levels, age and BMI (**Table 4**). The multivariate logistic regression analysis showed that for younger women (age<30 years old), regardless of their BMI and ovarian reserves, the GnRH agonist long-acting protocol was more likely to elicit live births than the antagonist protocol, although the difference was not statistically significant. However, among women who were above 30 years old and who had normal ovarian reserves (3ng/ml<AMH<6ng/ml) and variable BMI, the abilities of the two protocols to elicit live births may differ significantly. For women who had AMH levels from 3ng/ml to 6ng/ml (3ng/ml<AMH<6ng/ml), were aged ≥ 30 years old and had BMI ≥ 24kg/m^2^, the GnRH agonist long-acting protocol was more likely to have live births than the antagonist protocol [OR (95%CI), 2.13(1.19,3.80)]; while among the women with normal ovarian reserves, were aged ≥30 years old and had BMI < 24kg/m^2^, the chances to have live births were similar between the two protocol groups [OR (95%CI),0.99(0.72,1.37)]. Among women with AMH ≤ 3ng/ml, aged ≥ 30 years old and with BMI < 24kg/m^2^, the GnRH agonist long-acting protocol had a higher possibility to live births than the antagonist protocol [OR (95%CI), 1.41(1.05,1.91)]. Interestingly, for women with AMH ≤ 3ng/ml, age ≥ 30 years old and BMI≥ 24kg/m^2^, the GnRH agonist long-acting protocol had a lower possibility of live births the antagonist protocol [OR (95%CI), 0.54(0.32,0.90)]. Among the women who had AMH level ≥ 6ng/ml, aged ≥ 30 years old and had BMI < 24kg/m^2^, the possibilities to have live births were similar between the two protocols [OR (95%CI), 1.68(0.81,3.51)] However, among the women who had AMH level ≥ 6ng/ml, aged ≥ 30 years old and with BMI≥ 24kg/m^2^, the GnRH agonist long-acting protocol had a lower possibility of eliciting live birth than the antagonist protocol [OR (95%CI), 0.51(0.10,2.55)]. Before matching, and after adjusting potential confounding factors, the multivariate logistic regression analysis showed that for younger women (age<30 years old), who had normal ovarian reserves and with BMI < 24kg/m^2^, the GnRH agonist long-acting protocol was more likely to elicit live births than the antagonist protocol [OR (95%CI),1.58(1.16, 2.16)] (**Supplemental Table 1**).

**Table 4:** Multivariable logistic regression analysis of live birth rate of the GnRH agonist long-acting protocol and GnRH antagonist protocol for women with different AMH, Age and BMI (after PS matching) (the GnRH antagonist protocol group as a reference).

		after matching			
		Adjusted OR (95% CI)	P	Adjusted OR (95% CI)	P
		BMI<24.0kg/m2		BMI≥24.0kg/m2	
age<30year					
	AMH ≤ 3ng/ml	1.02 (0.68,1.54)	0.909	1.43 (0.68,2.98)	0.342
	3ng/ml <AMH<6ng/ml	1.38 (0.97,1.97)	0.072	1.06 (0.60,1.89)	0.842
	AMH≥6ng/ml	1.42 (0.75,2.69)	0.286	1.02 (0.19,5.35)	0.985
age≥30year					
	AMH ≤ 3ng/ml	1.41 (1.05,1.91)	0.024*	0.54 (0.32,0.90)	0.018*
	3ng/ml <AMH<6ng/ml	0.99 (0.72,1.37)	0.964	2.13 (1.19,3.80)	0.011*
	AMH≥6ng/ml	1.68 (0.81,3.51)	0.164	0.51 (0.10,2.55)	0.413

adjusting for confounders of female age, BMI, AMH, AFC, E2, FSH, LH, P, number of good quality embryos, total dose of Gn, duration of Gn.

^*^Statistically significant (P < 0.05).

## Discussion

Providing an individualized IVF-ET protocol, *via* individual characteristics, so as to maximize the rate of pregnancy and live births while reducing OHSS and adverse pregnancy outcomes, is still a big challenge in clinical medicine. In this study, we first analyzed the variables of the participants without any special stratification; and observed that the GnRH agonist long-acting protocol group had higher live birth rates, biochemical pregnancy rates and clinical pregnancy rates than the antagonist protocol group (**Tables 2**, **3**). This is consistent with the findings of other studies (4, 30) which showed that in the fresh cycle, the GnRH agonist long-acting protocol group had a higher clinical pregnancy rate and implantation rate than the GnRH antagonist protocol group. The mRNA and protein levels of HOXA10, MEIS1 and LIF, which are markers of uterine development and endometrial receptivity (31, 32), were found to be higher in the GnRH agonist long-acting protocol group than in the antagonist protocol group. This indicates that the GnRH agonist long-acting protocol, unlike the antagonist protocol, may have a less association with the impairment of the patients’ endometrial receptivity. In addition, we found that the GnRH agonist long-acting protocol was associated with a higher risk of OHSS (4.57% vs 1.91%), which is consistent with Toftager et al’s results (33). These findings indicate that the GnRH agonist long-acting protocol, rather than the GnRH antagonist protocol, may be more beneficial to women who undergo ART therapy.

To date, there is no single COS solution that works for all infertile women. Zhang et al. (34) indicated that the choice of COS protocol is highly dependent on ovarian reserve and age. Marci et al. (35) reported that high BMI could impair the ovarian response to exogenous gonadotropins. However, it is not a common practice to combine these factors to select a COS protocol for infertile women. Therefore, to explore whether women with different characteristics are more suitable for any protocol, we divided the study population into several groups according to the ages, AMH levels and BMI of the study participants. We found that among women with normal ovarian reserve, BMI < 24kg/m^2^ and age <30 years old, the GnRH agonist long-acting protocol was associated with a higher possibility of having live birth than that of the GnRH antagonist protocol [OR (95%CI),1.58(1.16,2.16)] (**Supplemental Table 1**). Grow et al. (36) reported that good-prognosis patients had higher live birth rate with the GnRH agonist long-acting protocol than with the antagonist protocol [OR (95%CI),1.13(1.03,1.25)]. The results of this study are consistent with our findings. Additionally, in overweight women (BMI ≥ 24 kg/m2) with normal ovarian reserve, the women aged ≥ 30 years old had higher live birth rates with the GnRH agonist long-acting protocol than with the antagonist protocol [OR (95%CI), 2.13(1.19,3.80)]. Also, our results showed that a higher number of oocytes was retrieved in the GnRH agonist long-acting protocol group than in the antagonist protocol group. Since a decline in the number of oocyte as well as the increase of age, old age (37) and embryo aneuploidy (38) are crucial factors of infertility, the GnRH agonist long-acting protocol is recommended for infertile women with normal ovarian reserve, who have BMI<24kg/m2 and are aged <30 years old as well as those who have normal ovarian reserve have BMI ≥ 24kg/m2 and are aged ≥ 30 years.

Further, in women with normal ovarian reserve (3ng/ml < AMH < 6ng/ml), with BMI < 24kg/m2 and are aged ≥30 years old or with BMI ≥ 24 kg/m2 and with ages < 30 years old, the possibilities to have live births were similar between the two protocols [OR (95%CI), 0.99(0.72,1.37)], [OR (95%CI), 1.06(0.60,1.89)]. Our results are consistent with that of a meta-analysis (9) which showed no difference between the agonist protocol group and the antagonist protocol group of women with normal ovarian reserves (OR [95% CI] = 0.95 [0.74, 1.09], P = 0.27). Al-Inany et al. [35] found that compared to the GnRH agonist long-acting protocol, the antagonist protocol significantly reduced the incidence of any grade of OHSS (OR 0.61, 95% C 0.51 to 0.72; 36 RCTs, n = 7944, I2 = 31%, moderate quality evidence) without affecting the live birth rate (OR 1.02, 95% CI 0.85 to 1.23; 12 RCTs, n = 2303, I2 = 27%, moderate quality evidence). Therefore, the antagonist protocol is recommended for infertile women with normal ovarian reserve, with BMI < 24kg/m^2^ and with ages ≥30 years or with BMI ≥ 24kg/m^2^ and with ages < 30 years.

Other studies (33, 39–41) have reported that the GnRH antagonist protocol is safer for women with a low and high ovarian reserve, just that live birth rates are similar in both protocols. Our study with larger sample size further revealed that, regardless of age and BMI, among women with relatively high ovarian reserve (AMH ≥ 6 ng/ml), the two protocols had similar live birth rates. Particularly, in women with relatively high ovarian reserve (AMH ≥ 6 ng/ml), with BMI ≥ 24kg/m2 and have ages ≥30 years, the possibility of getting live birth in the GnRH agonist protocol was lower although the difference was not significant [OR (95%CI), 0.54(0.32,0.90)]. Moreover, among younger (age <30 years) women with relatively low ovarian reserve (AMH≤ 3ng/ml), regardless of BMI, the live birth rate was similar in the two protocols. Therefore, the GnRH antagonist protocol is strongly recommended for women with the above characteristics.

Li et al. (10) reported that among women in POSEIDON group 4 of advanced age and have diminished ovarian reserves, the GnRH agonist long-acting protocol and the antagonist protocol achieved comparable live birth rates. However, our study found that among the women with relatively low ovarian reserve (AMH ≤ 3ng/ml), with ages ≥ 30 years old and with BMI < 24kg/m2, the GnRH agonist long-acting protocol was more likely to have live births than the antagonist protocol [OR (95%CI), 1.41(1.05,1.91)]; while among women with relatively low ovarian reserve (AMH ≤ 3ng/ml), with age ≥ 30 years old and with BMI ≥ 24kg/m2, the GnRH agonist long-acting protocol had a lower possibility of live birth than the antagonist protocol [OR (95%CI), 0.54(0.32,0.90)]. These indicate that BMI is a vital factor to be considered in a personalized COS protocol. Unfortunately, to the best of our knowledge, there have been no studies comparing the GnRH agonist long-acting protocol and the GnRH antagonist protocol in women who have low ovarian reserve and who have different BMIs. Rabinson et al. (42) showed that in general women with BMI < 25kg/m2, the GnRH agonist protocol had a higher pregnancy rate. Although the ovarian reserve of women included in the study was not selected, the trend of their results was consistent with ours. These findings show that the GnRH agonist long-acting protocol may be more suitable for women with relatively low ovarian reserve (AMH ≤ 3ng/ml), with ages ≥ 30 years old and with BMI < 24kg/m2. Nevertheless, among women with relatively low ovarian reserve (AMH ≤ 3ng/ml), with age ≥ 30 years old and with BMI ≥ 24kg/m^2^, the GnRH antagonist protocol is recommended since it can help avoid the excessive suppression of the pituitary-gonadal axis and the concentrations of endogenous FSH and LH (43).

To our knowledge, this is the first study to compare the live birth rates of the GnRH agonist long-acting protocol and antagonist protocol in women with different characteristics by combining BMI with ovarian reserve markers. In spite of all the efforts to control bias, this study is inherently limited by the review of a retrospectively collected data set. In addition, this study did not follow up to the frozen embryo cycle, and could not provide relevant indicators such as cumulative live birth rate.

## Conclusion

Among infertile women who receive fresh embryo transfer after the first IVF treatment, the GnRH agonist long-acting protocol is recommended for women with normal ovarian reserve (3ng/ml < AMH < 6ng/ml), with BMI<24 kg/m^2^ and with ages<30 years, and for those with normal ovarian reserve (3ng/ml < AMH < 6ng/ml), with BMI ≥ 24 kg/m^2^ and are aged above 30 years. It is also recommended for women with BMI < 24kg/m^2^ and with ages<30 years whose AMH levels are ≤ 3ng/ml. However, among the remaining infertile women in the cohort, the antagonist protocol may suite them because of the lower incidence of ovarian hyperstimulation syndrome, duration and dosage of Gn. Taken together, our results may provide a personalized recommendation in COS protocol selection. The recommendation of two protocols for women in different characters is shown in **Supplemental Table 2**.

## Data Availability Statement

The data used in this article were obtained from the Chengdu Xinan Gynecology Hospital and Chengdu Jinjiang Hospital for Women’s and Children’s Health by request. Upon data request, the corresponding author would obtain permission from the Chengdu Xinan Gynecology Hospital and Chengdu Jinjiang Hospital for Women’s and Children’s Health before sharing them.

## Author Contributions

M-XC contributed to study design, data collection, statistical analysis and drafting of the manuscript. QW assisted with data collection and interpretation. X-JT reviewed the analyzed results. Z-HZ reviewed the analyzed results and revised the manuscript. X-QM, TL, QF, YJ, X-YL, L-HG, LZ and QW provided ART-related clinical theory and technical support. Y-BD and Enoch Appiah Adu-Gyamfi critically revised the manuscript. All authors contributed to the article and approved the submitted version.

## Funding

This study was funded by the the National Natural Science Foundation of China (Grant No. 81971391), National Key Research and Development Program of China (2017YFC1002001), and the Chongqing Science and Technology Bureau (Grant No. cstc2019jxjl130030).

## Conflict of Interest

The authors have no conflict of interest to declare.

## Publisher’s Note

All claims expressed in this article are solely those of the authors and do not necessarily represent those of their affiliated organizations, or those of the publisher, the editors and the reviewers. Any product that may be evaluated in this article, or claim that may be made by its manufacturer, is not guaranteed or endorsed by the publisher.
