# Supplementary material for: An Individualized Recommendation for Controlled Ovary Stimulation Protocol in Women Who Received the GnRH Agonist Long-Acting Protocol or the GnRH Antagonist Protocol: A Retrospective Cohort Study
Source: Front Endocrinol (Lausanne). 2022 Jul 22;13:899000. doi: 10.3389/fendo.2022.899000 (PMC9355571; doi:10.3389/fendo.2022.899000)
Supplement: Supplementary file 1 [file Table_1.pdf]

Supplemental Table1.Multivariable logistic regression analysis of live birth rate of the GnRH agonist long-acting protocol and GnRH antagonist protocol for women with different AMH, Age and BMI (before PS matching) （the GnRH antagonist protocol group as a reference）

		Before matching			
		Adjusted OR (95% CI)	P	Adjusted OR (95% CI)	P
		BMI<24.0kg/m2		BMI⩾24.0kg/m2	
age<30year					
	AMH≤3ng/ml	1.12(0.79,1.58)	0.521	1.62(0.87,3.04)	0.131
	3ng/ml <AMH<6ng/ml	1.58(1.16,2.16)	0.004*	1.07(0.63,1.84)	0.799
	AMH≥6ng/ml	1.30(0.74,2.28)	0.363	0.47(0.11,1.98)	0.300
age⩾30year					
	AMH≤3ng/ml	1.27(0.98,1.65)	0.076	0.60(0.39,0.94)	0.025*
	3ng/ml <AMH<6ng/ml	1.06(0.79,1.42)	0.698	1.76(1.04,2.98)	0.034*
	AMH≥6ng/ml	1.67(0.88,3.17)	0.118	0.39(0.10,1.59)	0.189

adjusting for confounders of female age, BMI, AMH, AFC, E2, FSH, LH, P, number of good quality embryos, total dose of Gn, duration of Gn.

*Statistically significant ($P < 0.05$).

Supplemental Table2.

The recommendation of the GnRH agonist long-acting protocol and the GnRH antagonist protocol for infertile women who receive fresh embryo transfer after the first IVF/ICSI treatment.

		BMI<24.0kg/m2	BMI⩾24.0kg/m2
age<30year			
	AMH≤3ng/ml	GnRH antagonist protocol	GnRH antagonist protocol
	3ng/ml <AMH<6ng/ml	GnRH agonist long-acting protocol+	GnRH antagonist protocol
	AMH≥6ng/ml	GnRH antagonist protocol	GnRH antagonist protocol
age⩾30year			
	AMH≤3ng/ml	GnRH agonist long-acting protocol+	GnRH antagonist protocol+
	3ng/ml <AMH<6ng/ml	GnRH antagonist protocol	GnRH agonist long-acting protocol+
	AMH≥6ng/ml	GnRH antagonist protocol	GnRH antagonist protocol

+ represent strong recommendation
